# 2-(Methoxy­carbon­yl)quinolinium tetra­chlorido(quinoline-2-carboxyl­ato-κ^2^ *N*,*O*)stannate(IV) methanol solvate

**DOI:** 10.1107/S1600536810008561

**Published:** 2010-03-10

**Authors:** Marzieh Vafaee, Mostafa M. Amini, Seik Weng Ng

**Affiliations:** a Department of Chemistry, General Campus, Shahid Beheshti University, Tehran 1983963113, Iran; b Department of Chemistry, University of Malaya, 50603 Kuala Lumpur, Malaysia

## Abstract

In the title salt, (C_11_H_10_NO_2_)[SnCl_4_(C_10_H_6_NO_2_)]·CH_3_OH, the Sn atom is chelated by the quinolincarboxyl­ate unit and it exists in a distorted octa­hedral coordination geometry. The cation is linked to the solvent mol­ecule by an N—H⋯O hydrogen bond; the solvent mol­ecule is linked to the anion by an O—H⋯O hydrogen bond.

## Related literature

For the structure of 2-(ethoxy­carbon­yl)quinolinium *n*-butyl­trichlorido(quinolin-2-carboxyl­ato)stannate(IV), see: Wang *et al.* (2008 ▶).
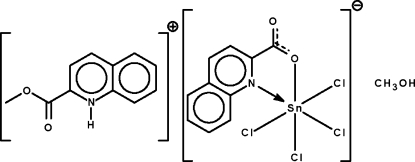


## Experimental

### 

#### Crystal data

(C_11_H_10_NO_2_)[SnCl_4_(C_10_H_6_NO_2_)]·CH_4_O
*M* *_r_* = 652.89
Monoclinic, 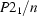

*a* = 8.4109 (4) Å
*b* = 33.2728 (16) Å
*c* = 10.0241 (5) Å
β = 112.8616 (6)°
*V* = 2584.9 (2) Å^3^
*Z* = 4
Mo *K*α radiation
μ = 1.44 mm^−1^
*T* = 293 K
0.35 × 0.25 × 0.15 mm

#### Data collection

Bruker SMART APEX diffractometer
Absorption correction: multi-scan (*SADABS*; Sheldrick, 1996 ▶) *T*_min_ = 0.633, *T*_max_ = 0.813
24727 measured reflections
5924 independent reflections
5288 reflections with *I* > 2σ(*I*)
*R*_int_ = 0.026

#### Refinement

*R*[*F* ^2^ > 2σ(*F* ^2^)] = 0.039
*wR*(*F* ^2^) = 0.104
*S* = 1.17
5924 reflections
317 parameters
2 restraints
H atoms treated by a mixture of independent and constrained refinement
Δρ_max_ = 0.38 e Å^−3^
Δρ_min_ = −1.15 e Å^−3^

### 

Data collection: *APEX2* (Bruker, 2009 ▶); cell refinement: *SAINT* (Bruker, 2009 ▶); data reduction: *SAINT*; program(s) used to solve structure: *SHELXS97* (Sheldrick, 2008 ▶); program(s) used to refine structure: *SHELXL97* (Sheldrick, 2008 ▶); molecular graphics: *X-SEED* (Barbour, 2001 ▶); software used to prepare material for publication: *publCIF* (Westrip, 2010 ▶).

## Supplementary Material

Crystal structure: contains datablocks global, I. DOI: 10.1107/S1600536810008561/bt5208sup1.cif

Structure factors: contains datablocks I. DOI: 10.1107/S1600536810008561/bt5208Isup2.hkl

Additional supplementary materials:  crystallographic information; 3D view; checkCIF report

## Figures and Tables

**Table 1:** Hydrogen-bond geometry (Å, °)

*D*—H⋯*A*	*D*—H	H⋯*A*	*D*⋯*A*	*D*—H⋯*A*
O5—H5⋯O2	0.84 (1)	1.95 (1)	2.785 (4)	176 (5)
N3—H3⋯O5	0.86 (1)	1.85 (2)	2.693 (4)	166 (4)

**Fig. 1.**
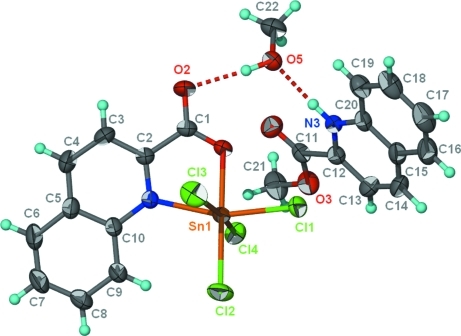
Anisotropic displacement ellipsoid plot (Barbour, 2001) of [C11H10NO2][SnCl4(C10H6NO2)].CH3OH; ellipsoids are drawn at the 50% probability level and H atoms are of arbitrary radius. Hydrogen bonds are drawn as dashed lines.

## supplementary crystallographic information

### Comment

Quinolin-2-carboxylic acid forms a number of compounds with organotin(IV) systems in which the deprotonated anion *N*,*O*-chelates to the tin atom. Such organotin carboxylates are conveniently synthesized by the reaction of an organotin chloride with the sodium salt of the carboxylic acid. Curiously, the reaction of sodium quinolin-2-carboxylate with *n*-butyltin trichloride furnishes the *n*-butyltrichlorido(quinolincarboxylato)stannate anion, whose charge is balanced by an ethyl quinoliniumcarboxylate cation (Wang *et al.*, 2008). The ethyl unit arises from the ethanol solvent used in the synthesis.

In our hands, the reaction of quinolin-2-carboxylic acid with stannic chloride has yielded a similar salt, which crystallizes as a methanol solvate (Scheme I, Fig. 1). The solvent is also involved in the esterification of the acid to furnish the cation. The cation is linked to the solvent molecule by an N–H···O hydrogen bond; the solvent molecule is linked to the anion by an O–H···O hydrogen bond.

### Experimental

Stannic chloride pentahydrate (1 mmol, 0.350 g) and quinaldic acid (2 mmol, 0.173 g) were dissolved in dry methanol. The solvent was allowed to evaporate to afford colorless crystals after 1 week.

### Refinement

Carbon-bound H-atoms were placed in calculated positions (C—H 0.93 to 0.96 Å) and were included in the refinement in the riding model approximation, with *U*(H) set to 1.2 to 1.5*U*(C). The nitrogen- and oxygen-bound ones were located in a difference Fourier map, and were refined isotropically with distance restraints of N–H = O–H 0.86±0.01 Å.

### Crystal data

**Table:**

(C_11_H_10_NO_2_)[SnCl_4_(C_10_H_6_NO_2_)]·CH_4_O	*F*(000) = 1296
*M**_r_* = 652.89	*D*_x_ = 1.678 Mg m^−^^3^
Monoclinic, *P*2_1_/*n*	Mo *K*α radiation, λ = 0.71073 Å
Hall symbol: -P 2yn	Cell parameters from 9921 reflections
*a* = 8.4109 (4) Å	θ = 2.3–28.1°
*b* = 33.2728 (16) Å	µ = 1.44 mm^−^^1^
*c* = 10.0241 (5) Å	*T* = 293 K
β = 112.8616 (6)°	Wedge, colorless
*V* = 2584.9 (2) Å^3^	0.35 × 0.25 × 0.15 mm
*Z* = 4	

### Data collection

**Table:**

Bruker SMART APEX diffractometer	5924 independent reflections
Radiation source: fine-focus sealed tube	5288 reflections with *I* > 2σ(*I*)
graphite	*R*_int_ = 0.026
ω scans	θ_max_ = 27.5°, θ_min_ = 1.2°
Absorption correction: multi-scan (*SADABS*; Sheldrick, 1996)	*h* = −10→10
*T*_min_ = 0.633, *T*_max_ = 0.813	*k* = −43→42
24727 measured reflections	*l* = −13→13

### Refinement

**Table:**

Refinement on *F*^2^	Primary atom site location: structure-invariant direct methods
Least-squares matrix: full	Secondary atom site location: difference Fourier map
*R*[*F*^2^ > 2σ(*F*^2^)] = 0.039	Hydrogen site location: inferred from neighbouring sites
*wR*(*F*^2^) = 0.104	H atoms treated by a mixture of independent and constrained refinement
*S* = 1.17	*w* = 1/[σ^2^(*F*_o_^2^) + (0.0423*P*)^2^ + 3.5411*P*] where *P* = (*F*_o_^2^ + 2*F*_c_^2^)/3
5924 reflections	(Δ/σ)_max_ = 0.001
317 parameters	Δρ_max_ = 0.38 e Å^−^^3^
2 restraints	Δρ_min_ = −1.15 e Å^−^^3^

### Fractional atomic coordinates and isotropic or equivalent isotropic displacement parameters (Å^2^)

**Table:**

	*x*	*y*	*z*	*U*_iso_*/*U*_eq_	
Sn1	0.77625 (3)	0.625981 (7)	0.62161 (2)	0.03525 (9)	
Cl1	0.79773 (15)	0.56055 (3)	0.53317 (11)	0.0513 (2)	
Cl2	0.86453 (17)	0.60335 (4)	0.86654 (11)	0.0633 (3)	
Cl3	1.06377 (14)	0.64544 (4)	0.66027 (14)	0.0661 (3)	
Cl4	0.47849 (13)	0.61972 (3)	0.58488 (12)	0.0492 (2)	
O1	0.6997 (4)	0.64512 (8)	0.4078 (3)	0.0419 (6)	
O2	0.6522 (5)	0.69652 (10)	0.2599 (3)	0.0665 (9)	
O3	0.1333 (4)	0.58105 (10)	0.2844 (4)	0.0573 (8)	
O4	0.2708 (4)	0.62339 (9)	0.1913 (4)	0.0566 (8)	
O5	0.5646 (4)	0.63000 (9)	0.0768 (3)	0.0476 (6)	
N1	0.7356 (4)	0.69426 (9)	0.6318 (3)	0.0388 (7)	
N3	0.4799 (4)	0.56215 (9)	0.1803 (3)	0.0376 (6)	
C1	0.6875 (5)	0.68256 (12)	0.3810 (4)	0.0439 (9)	
C2	0.7133 (5)	0.71104 (11)	0.5058 (4)	0.0408 (8)	
C3	0.7086 (7)	0.75223 (13)	0.4836 (5)	0.0591 (12)	
H3A	0.6919	0.7626	0.3930	0.071*	
C4	0.7287 (7)	0.77719 (13)	0.5963 (5)	0.0638 (13)	
H4	0.7297	0.8049	0.5840	0.077*	
C5	0.7481 (6)	0.76113 (12)	0.7317 (5)	0.0505 (10)	
C6	0.7654 (7)	0.78527 (14)	0.8525 (6)	0.0676 (14)	
H6	0.7710	0.8131	0.8455	0.081*	
C7	0.7741 (8)	0.76872 (16)	0.9772 (6)	0.0747 (16)	
H7	0.7831	0.7851	1.0550	0.090*	
C8	0.7696 (8)	0.72689 (16)	0.9909 (5)	0.0726 (15)	
H8	0.7743	0.7157	1.0774	0.087*	
C9	0.7581 (7)	0.70231 (14)	0.8775 (5)	0.0573 (12)	
H9	0.7571	0.6746	0.8882	0.069*	
C10	0.7480 (5)	0.71862 (11)	0.7467 (4)	0.0421 (8)	
C11	0.2513 (5)	0.59121 (12)	0.2346 (4)	0.0423 (8)	
C12	0.3641 (5)	0.55578 (11)	0.2379 (4)	0.0412 (8)	
C13	0.3513 (6)	0.51870 (13)	0.2949 (5)	0.0530 (10)	
H13	0.2686	0.5143	0.3337	0.064*	
C14	0.4601 (7)	0.48872 (13)	0.2940 (5)	0.0599 (12)	
H14	0.4528	0.4639	0.3339	0.072*	
C15	0.5835 (6)	0.49474 (12)	0.2337 (4)	0.0503 (10)	
C16	0.7009 (7)	0.46501 (15)	0.2295 (6)	0.0681 (14)	
H16	0.7010	0.4399	0.2703	0.082*	
C17	0.8142 (7)	0.47287 (17)	0.1659 (6)	0.0739 (16)	
H17	0.8914	0.4531	0.1638	0.089*	
C18	0.8150 (6)	0.51091 (17)	0.1030 (6)	0.0671 (14)	
H18	0.8917	0.5156	0.0584	0.081*	
C19	0.7058 (5)	0.54069 (14)	0.1064 (5)	0.0524 (10)	
H19	0.7081	0.5656	0.0655	0.063*	
C20	0.5903 (5)	0.53305 (11)	0.1727 (4)	0.0418 (8)	
C21	0.0198 (7)	0.61294 (16)	0.2925 (7)	0.0707 (14)	
H21A	−0.0661	0.6020	0.3231	0.106*	
H21B	−0.0355	0.6251	0.1989	0.106*	
H21C	0.0859	0.6328	0.3609	0.106*	
C22	0.4323 (7)	0.64239 (17)	−0.0535 (5)	0.0680 (13)	
H22A	0.3222	0.6360	−0.0511	0.102*	
H22B	0.4445	0.6288	−0.1334	0.102*	
H22C	0.4400	0.6709	−0.0649	0.102*	
H3	0.490 (6)	0.5852 (7)	0.145 (4)	0.051 (13)*	
H5	0.589 (7)	0.6495 (10)	0.135 (4)	0.069 (16)*	

### Atomic displacement parameters (Å^2^)

**Table:**

	*U*^11^	*U*^22^	*U*^33^	*U*^12^	*U*^13^	*U*^23^
Sn1	0.03944 (14)	0.03703 (14)	0.02901 (13)	0.00101 (10)	0.01300 (10)	0.00026 (9)
Cl1	0.0677 (7)	0.0394 (5)	0.0486 (5)	0.0063 (4)	0.0244 (5)	−0.0038 (4)
Cl2	0.0838 (8)	0.0711 (7)	0.0352 (5)	0.0275 (6)	0.0232 (5)	0.0130 (5)
Cl3	0.0402 (6)	0.0898 (9)	0.0675 (7)	−0.0076 (5)	0.0202 (5)	−0.0077 (6)
Cl4	0.0413 (5)	0.0480 (5)	0.0587 (6)	−0.0016 (4)	0.0200 (4)	0.0033 (4)
O1	0.0560 (16)	0.0409 (14)	0.0290 (12)	−0.0004 (12)	0.0168 (11)	−0.0010 (10)
O2	0.113 (3)	0.0531 (18)	0.0342 (15)	−0.0056 (18)	0.0291 (17)	0.0064 (13)
O3	0.0554 (18)	0.0594 (19)	0.071 (2)	−0.0112 (14)	0.0394 (16)	−0.0129 (15)
O4	0.067 (2)	0.0469 (17)	0.067 (2)	0.0100 (14)	0.0382 (17)	0.0064 (14)
O5	0.0541 (17)	0.0469 (16)	0.0389 (14)	−0.0048 (13)	0.0151 (13)	−0.0037 (12)
N1	0.0468 (17)	0.0349 (16)	0.0379 (16)	−0.0054 (13)	0.0199 (14)	−0.0032 (12)
N3	0.0431 (17)	0.0348 (16)	0.0344 (15)	−0.0011 (13)	0.0147 (13)	−0.0012 (12)
C1	0.054 (2)	0.046 (2)	0.0339 (18)	−0.0029 (17)	0.0194 (17)	0.0027 (15)
C2	0.050 (2)	0.0383 (19)	0.0363 (18)	−0.0022 (16)	0.0187 (16)	0.0012 (15)
C3	0.089 (4)	0.043 (2)	0.050 (2)	−0.006 (2)	0.032 (2)	0.0099 (19)
C4	0.096 (4)	0.034 (2)	0.067 (3)	−0.006 (2)	0.039 (3)	−0.002 (2)
C5	0.064 (3)	0.036 (2)	0.058 (2)	−0.0072 (18)	0.031 (2)	−0.0069 (18)
C6	0.096 (4)	0.041 (2)	0.078 (3)	−0.011 (2)	0.046 (3)	−0.021 (2)
C7	0.111 (5)	0.063 (3)	0.070 (3)	−0.015 (3)	0.057 (3)	−0.030 (3)
C8	0.114 (5)	0.069 (3)	0.055 (3)	−0.018 (3)	0.055 (3)	−0.016 (2)
C9	0.089 (3)	0.045 (2)	0.051 (2)	−0.012 (2)	0.042 (2)	−0.0110 (19)
C10	0.052 (2)	0.0368 (19)	0.043 (2)	−0.0080 (16)	0.0240 (18)	−0.0077 (15)
C11	0.043 (2)	0.046 (2)	0.0396 (19)	−0.0044 (16)	0.0181 (17)	−0.0102 (16)
C12	0.045 (2)	0.040 (2)	0.0386 (19)	−0.0048 (16)	0.0167 (16)	−0.0045 (15)
C13	0.064 (3)	0.044 (2)	0.056 (2)	−0.010 (2)	0.028 (2)	0.0029 (19)
C14	0.083 (3)	0.036 (2)	0.058 (3)	−0.007 (2)	0.024 (2)	0.0033 (19)
C15	0.059 (3)	0.037 (2)	0.043 (2)	0.0062 (18)	0.0070 (19)	−0.0042 (16)
C16	0.078 (3)	0.045 (3)	0.064 (3)	0.018 (2)	0.009 (3)	−0.006 (2)
C17	0.062 (3)	0.070 (3)	0.073 (3)	0.028 (3)	0.008 (3)	−0.017 (3)
C18	0.046 (3)	0.083 (4)	0.068 (3)	0.009 (2)	0.018 (2)	−0.024 (3)
C19	0.045 (2)	0.058 (3)	0.054 (2)	0.0022 (19)	0.0180 (19)	−0.011 (2)
C20	0.042 (2)	0.0378 (19)	0.0391 (19)	0.0025 (15)	0.0087 (16)	−0.0082 (15)
C21	0.061 (3)	0.075 (3)	0.093 (4)	−0.003 (2)	0.048 (3)	−0.024 (3)
C22	0.086 (4)	0.077 (3)	0.043 (2)	0.001 (3)	0.027 (2)	0.001 (2)

### Geometric parameters (Å, °)

**Table:**

Sn1—O1	2.083 (2)	C7—C8	1.400 (7)
Sn1—N1	2.305 (3)	C7—H7	0.9300
Sn1—Cl1	2.3838 (10)	C8—C9	1.373 (6)
Sn1—Cl3	2.3853 (11)	C8—H8	0.9300
Sn1—Cl2	2.3937 (10)	C9—C10	1.391 (6)
Sn1—Cl4	2.3947 (10)	C9—H9	0.9300
O1—C1	1.270 (5)	C11—C12	1.505 (5)
O2—C1	1.223 (4)	C12—C13	1.382 (6)
O3—C11	1.317 (5)	C13—C14	1.356 (7)
O3—C21	1.451 (6)	C13—H13	0.9300
O4—C11	1.190 (5)	C14—C15	1.404 (7)
O5—C22	1.409 (6)	C14—H14	0.9300
O5—H5	0.842 (10)	C15—C16	1.410 (6)
N1—C2	1.325 (5)	C15—C20	1.425 (6)
N1—C10	1.379 (5)	C16—C17	1.362 (8)
N3—C12	1.328 (5)	C16—H16	0.9300
N3—C20	1.364 (5)	C17—C18	1.415 (8)
N3—H3	0.860 (10)	C17—H17	0.9300
C1—C2	1.517 (5)	C18—C19	1.360 (6)
C2—C3	1.387 (6)	C18—H18	0.9300
C3—C4	1.358 (6)	C19—C20	1.397 (6)
C3—H3A	0.9300	C19—H19	0.9300
C4—C5	1.408 (6)	C21—H21A	0.9600
C4—H4	0.9300	C21—H21B	0.9600
C5—C6	1.413 (6)	C21—H21C	0.9600
C5—C10	1.423 (5)	C22—H22A	0.9600
C6—C7	1.342 (7)	C22—H22B	0.9600
C6—H6	0.9300	C22—H22C	0.9600
			
O1—Sn1—N1	75.66 (10)	C8—C9—C10	120.5 (4)
O1—Sn1—Cl1	86.24 (8)	C8—C9—H9	119.8
N1—Sn1—Cl1	161.86 (8)	C10—C9—H9	119.8
O1—Sn1—Cl3	88.44 (8)	N1—C10—C9	121.0 (3)
N1—Sn1—Cl3	83.31 (9)	N1—C10—C5	119.9 (3)
Cl1—Sn1—Cl3	95.15 (4)	C9—C10—C5	119.0 (4)
O1—Sn1—Cl2	179.45 (8)	O4—C11—O3	126.9 (4)
N1—Sn1—Cl2	104.89 (8)	O4—C11—C12	122.5 (4)
Cl1—Sn1—Cl2	93.21 (4)	O3—C11—C12	110.6 (3)
Cl3—Sn1—Cl2	91.59 (5)	N3—C12—C13	120.7 (4)
O1—Sn1—Cl4	88.95 (8)	N3—C12—C11	115.3 (3)
N1—Sn1—Cl4	85.89 (8)	C13—C12—C11	123.9 (4)
Cl1—Sn1—Cl4	95.12 (4)	C14—C13—C12	119.5 (4)
Cl3—Sn1—Cl4	169.21 (4)	C14—C13—H13	120.2
Cl2—Sn1—Cl4	91.12 (4)	C12—C13—H13	120.2
C1—O1—Sn1	119.0 (2)	C13—C14—C15	120.8 (4)
C11—O3—C21	116.3 (4)	C13—C14—H14	119.6
C22—O5—H5	108 (4)	C15—C14—H14	119.6
C2—N1—C10	119.0 (3)	C16—C15—C14	123.8 (5)
C2—N1—Sn1	110.0 (2)	C16—C15—C20	118.0 (5)
C10—N1—Sn1	130.7 (2)	C14—C15—C20	118.2 (4)
C12—N3—C20	122.7 (3)	C17—C16—C15	120.3 (5)
C12—N3—H3	122 (3)	C17—C16—H16	119.8
C20—N3—H3	116 (3)	C15—C16—H16	119.8
O2—C1—O1	123.4 (4)	C16—C17—C18	120.4 (4)
O2—C1—C2	118.9 (4)	C16—C17—H17	119.8
O1—C1—C2	117.6 (3)	C18—C17—H17	119.8
N1—C2—C3	123.6 (4)	C19—C18—C17	121.3 (5)
N1—C2—C1	116.4 (3)	C19—C18—H18	119.3
C3—C2—C1	120.0 (3)	C17—C18—H18	119.3
C4—C3—C2	119.0 (4)	C18—C19—C20	118.7 (5)
C4—C3—H3A	120.5	C18—C19—H19	120.6
C2—C3—H3A	120.5	C20—C19—H19	120.6
C3—C4—C5	120.0 (4)	N3—C20—C19	120.8 (4)
C3—C4—H4	120.0	N3—C20—C15	118.0 (4)
C5—C4—H4	120.0	C19—C20—C15	121.2 (4)
C4—C5—C6	123.1 (4)	O3—C21—H21A	109.5
C4—C5—C10	118.3 (4)	O3—C21—H21B	109.5
C6—C5—C10	118.6 (4)	H21A—C21—H21B	109.5
C7—C6—C5	121.0 (4)	O3—C21—H21C	109.5
C7—C6—H6	119.5	H21A—C21—H21C	109.5
C5—C6—H6	119.5	H21B—C21—H21C	109.5
C6—C7—C8	120.3 (4)	O5—C22—H22A	109.5
C6—C7—H7	119.8	O5—C22—H22B	109.5
C8—C7—H7	119.8	H22A—C22—H22B	109.5
C9—C8—C7	120.5 (5)	O5—C22—H22C	109.5
C9—C8—H8	119.8	H22A—C22—H22C	109.5
C7—C8—H8	119.8	H22B—C22—H22C	109.5
			
N1—Sn1—O1—C1	8.4 (3)	Sn1—N1—C10—C9	−12.5 (6)
Cl1—Sn1—O1—C1	−170.3 (3)	C2—N1—C10—C5	−4.1 (6)
Cl3—Sn1—O1—C1	−75.1 (3)	Sn1—N1—C10—C5	168.4 (3)
Cl2—Sn1—O1—C1	−169 (9)	C8—C9—C10—N1	−179.7 (5)
Cl4—Sn1—O1—C1	94.5 (3)	C8—C9—C10—C5	−0.6 (7)
O1—Sn1—N1—C2	−10.0 (3)	C4—C5—C10—N1	2.4 (7)
Cl1—Sn1—N1—C2	−6.0 (5)	C6—C5—C10—N1	−178.4 (4)
Cl3—Sn1—N1—C2	80.1 (3)	C4—C5—C10—C9	−176.7 (5)
Cl2—Sn1—N1—C2	170.0 (2)	C6—C5—C10—C9	2.5 (7)
Cl4—Sn1—N1—C2	−100.0 (3)	C21—O3—C11—O4	−2.7 (6)
O1—Sn1—N1—C10	177.0 (3)	C21—O3—C11—C12	177.7 (4)
Cl1—Sn1—N1—C10	−179.0 (2)	C20—N3—C12—C13	0.6 (6)
Cl3—Sn1—N1—C10	−92.9 (3)	C20—N3—C12—C11	−179.0 (3)
Cl2—Sn1—N1—C10	−3.0 (3)	O4—C11—C12—N3	−4.2 (6)
Cl4—Sn1—N1—C10	87.0 (3)	O3—C11—C12—N3	175.5 (3)
Sn1—O1—C1—O2	176.1 (3)	O4—C11—C12—C13	176.2 (4)
Sn1—O1—C1—C2	−5.7 (5)	O3—C11—C12—C13	−4.1 (5)
C10—N1—C2—C3	2.8 (6)	N3—C12—C13—C14	1.0 (6)
Sn1—N1—C2—C3	−171.2 (4)	C11—C12—C13—C14	−179.4 (4)
C10—N1—C2—C1	−175.6 (3)	C12—C13—C14—C15	−1.1 (7)
Sn1—N1—C2—C1	10.4 (4)	C13—C14—C15—C16	179.8 (5)
O2—C1—C2—N1	174.0 (4)	C13—C14—C15—C20	−0.5 (7)
O1—C1—C2—N1	−4.2 (6)	C14—C15—C16—C17	178.4 (5)
O2—C1—C2—C3	−4.5 (6)	C20—C15—C16—C17	−1.3 (7)
O1—C1—C2—C3	177.3 (4)	C15—C16—C17—C18	−0.2 (8)
N1—C2—C3—C4	0.4 (8)	C16—C17—C18—C19	1.2 (8)
C1—C2—C3—C4	178.8 (4)	C17—C18—C19—C20	−0.5 (7)
C2—C3—C4—C5	−2.1 (8)	C12—N3—C20—C19	177.7 (4)
C3—C4—C5—C6	−178.5 (5)	C12—N3—C20—C15	−2.1 (5)
C3—C4—C5—C10	0.8 (8)	C18—C19—C20—N3	179.2 (4)
C4—C5—C6—C7	176.3 (6)	C18—C19—C20—C15	−1.1 (6)
C10—C5—C6—C7	−2.9 (8)	C16—C15—C20—N3	−178.3 (4)
C5—C6—C7—C8	1.3 (9)	C14—C15—C20—N3	2.0 (6)
C6—C7—C8—C9	0.7 (10)	C16—C15—C20—C19	2.0 (6)
C7—C8—C9—C10	−1.0 (9)	C14—C15—C20—C19	−177.8 (4)
C2—N1—C10—C9	175.0 (4)		

### Hydrogen-bond geometry (Å, °)

**Table:**

*D*—H···*A*	*D*—H	H···*A*	*D*···*A*	*D*—H···*A*
O5—H5···O2	0.84 (1)	1.95 (1)	2.785 (4)	176 (5)
N3—H3···O5	0.86 (1)	1.85 (2)	2.693 (4)	166 (4)
